# Learning to safely integrate generative artificial intelligence technology into travel medicine practice

**DOI:** 10.1093/jtm/taad149

**Published:** 2023-11-28

**Authors:** Gerard Thomas Flaherty

**Affiliations:** School of Medicine, University of Galway, Galway, Ireland; School of Medicine, International Medical University, Kuala Lumpur, Malaysia; Department of Clinical Tropical Medicine, Faculty of Tropical Medicine, Mahidol University, Bangkok, Thailand

**Keywords:** Pre-travel consultation, risk assessment, decision support tool, data mining, machine learning, smartphone application, digital technology

## Abstract

We have entered an exciting era where generative artificial intelligence is finding multiple applications in everyday life and scientific inquiry. This editorial explores the possibility of integrating this technology into the pre-travel consultation, but with careful consideration of its current capabilities, limitations and potential risks to patient safety.

According to the Turing test, named after the English computer scientist and cryptanalyst, Alan Turing, if a machine can engage in conversation with a human without being detected as a machine, it has demonstrated human intelligence.^1^ Given that the number of parameters in the large language model based artificial intelligence (AI) chatbot, ChatGPT-4, is estimated at 1.76 trillion and that of the human brain an impressive 700 trillion, it may be some time before the Turing test criteria are fulfilled by modern machines.^2^

The accelerated pace of development of generative artificial intelligence (GenAI) technology has been astounding, however. In the space of months, people have already become comfortable interacting with GenAI chatbots such as ChatGPT or Google Bard; academic journals are guiding authors about how to acknowledge the use of GenAI in their manuscripts; and this author has recently attended a seminar in his university entitled ‘AI in Higher Education: Transforming Learning and Maintaining Integrity’. The volume of publications in PubMed devoted to ChatGPT alone has ballooned from 4 in 2022 to 1589 as of 31 October 2023. The publicly expressed concerns of leading figures, including the United Nations Secretary-General, António Guterres, who pointed to the ‘enormous potential’ of GenAI ‘for good and evil at scale’, have led to calls to pause AI experiments to allow society to prepare for its titanic impact. As with all new technologies, there will be innovators and laggards. Travel medicine, being a young, dynamic discipline heavily dependent on the provision of accurate, evidence-based information, should be to the fore in discussions around the potential role for GenAI in clinical practice.

Ngiam and colleagues, publishing in this journal, compared the pre-travel health advice submitted by ChatGPT in response to a series of standard questions against guidance from reputable travel medicine expert sources.^3^ They found that ChatGPT generated concise responses based on open access data it had been trained on up to September 2021. The information given was comprehensive but lacked specificity and context, failing to take account of the individual traveller’s itinerary, cost considerations, previous vaccines and medical history. Reassuringly, it did recommend referral to a healthcare provider, however. The researchers postulate that ChatGPT may serve to improve uptake of pre-travel advice offered during a travel health consultation, surely a laudable objective. The accessibility of the tool is also a significant advantage, especially for last-minute travellers.

Though it has not been well studied to date by travel medicine researchers, there has been a tendency, especially among less experienced practitioners, for travel health risk assessment and advice to be somewhat generic and less individualised than might be desirable.^4^ The considerable diversity of traveller characteristics, their priorities^5^ and travel itineraries deserves a more nuanced approach. A recent Australian study concluded that reliable digital decision aids could be used to help inform travellers’ vaccine decisions.^6^ Web scraping has also been used to mine information about German travellers’ vaccination attitudes and behaviours.^7^ If GenAI chatbots are to be deployed effectively in the pre-clinic setting, in preparation of clients for their ensuing clinical consultation, then we must offer more personalised counselling to our travellers that builds on the basic information communicated by the chatbot. Rather than being threatened by this technology, we should embrace it as an opportunity to make more efficient use of the limited time available to us in the pre-travel health consultation. When booking their appointments, travellers could, for example, be actively encouraged to consult a GenAI chatbot with a standardised query relevant to their travel destinations (e.g. ‘what health precautions should a person travelling from Switzerland to India consider?’), and ideally to triangulate information from multiple chatbots.

GenAI chatbots could be used to overcome language barriers during the consultation by offering translation services and to educate travellers based on their individual health literacy levels. They may also be used to prevent decay of knowledge gained from the primary consultation and to issue useful reminders about healthy traveller behaviour either before or during the travel itself. GenAI can also be used to relay specific emergency healthcare information to travellers, such as the closest healthcare providers and emergency contact numbers. In time, it may be possible to integrate GenAI chatbots with electronic health records to streamline the documentation of the travel health consultation. Ultimately, clinical oversight must be maintained by the travel medicine provider who will be responsible for assuming medicolegal liability, although, for now, at least this remains a grey area. Travellers must be reminded to consult their clinician rather than relying solely on advice generated by a GenAI chatbot. Fortunately, such disclaimers are already provided at the end of chat outputs relating to travel health advice. A reassuring example from a recent query of mine stated, ‘It’s essential to note that while ChatGPT can provide valuable information and support, it should not replace the expertise and personalized care offered by travel medicine clinicians. It should be used as a complementary tool to enhance the overall quality of pre-travel health consultations.’ This seems like a very reasonable proposition.

The choice of which GenAI chatbot to consult is not a trivial one. ChatGPT, the tool used by Ngiam et al. in this journal,^3^ is not directly connected with the internet and relies on data up to September 2021 to generate its response outputs. The experimental GenAI tool Bard, on the other hand, is linked with Google’s vast search network, which gives it access to higher volumes of real-time, current information from the internet.^8^ Bard may also be more suitable in a travel medicine context as it specialises in processing more factual queries requiring precise responses. Future studies should compare these and other GenAI chatbots. This will undoubtedly become a fertile field for inquiry. Datasets used to train these algorithms include sources which have not undergone peer review, including social media data, and may therefore be subject to factual inaccuracies or biases. So-called hallucinations (confabulation may be a more apt term) are well recognised whereby AI chatbots produce plausible but unverified or false information particularly when they fail to find robust data, which has obvious implications for patient safety.^9^ Will our untrained travellers be attentive to such fabrications?

It is predicted that GenAI will continue to learn and refine its output based on training with ever larger and more reliable datasets. As a travel medicine community, we should engage with data scientists and computer programmers to ensure that specific, comprehensive training data are employed to train machine learning algorithms and that chatbots are kept updated with the latest epidemiological data relating to destination-specific infectious disease outbreaks and travel health advisories. The recent collaboration of the GeoSentinel Network with data scientists in the Netherlands is a promising example of such interdisciplinary dialogue. Measures of the impact of published peer-reviewed articles, including citation metrics and altmetrics, should allow AI chatbots to source the most robust information available.

It has been argued that large language models such as ChatGPT may not be appropriate for providing valid responses to medical queries since they lack both citations and transparent reasoning.^10^ Haverkamp et al. challenged ChatGPT to respond to the query ‘Is ChatGPT able to provide evidence-based medical information?’.^11^ After an initial evasive answer, it was pressed to provide a binary response, to which the definitive answer was ‘No’. Whether future iterations of this and other GenAI chatbots will be able to achieve the degree of transparency in sources and data selection necessary to be able to meet the standard of evidence-based medicine remains to be seen. As newer, more sophisticated GenAI chatbots come into general use, they should be cautiously evaluated to ensure that they do not threaten the safety of travellers by providing misleading or erroneous information. This author shares the optimism of Kwok and colleagues in their recent article in this journal when they asserted that artificial and human intelligence can collaborate to deliver a comprehensive travel health consultation to travellers.^12^ Whether machines will ever pass the Turing test remains to be seen.

## Data Availability

Data sharing is not applicable to this article as no new data were created or analysed in this study.
